# Supplementary material for: The Level and Limitations of Physical Activity in Elderly Patients with Diabetes
Source: J Clin Med. 2024 Oct 23;13(21):6329. doi: 10.3390/jcm13216329 (PMC11546819; doi:10.3390/jcm13216329)
Supplement: Supplementary file 1 [file jcm-13-06329-s001.zip › Supplementary File S1.pdf]

# Accompanying questionnaire

# Patient's ID: …………………………..

# Initials (first name, last name): ………………

**Demography and epidemiology**

1. **Race:** fill in if not European**:................ Based on the last recommendation we withdrow this point:**
2. **Your sex (biological):** Female Male
3. **Age** (full years - if your birthday is tomorrow, for example, please fill in the age you are today): ...............
4. **Level of education**: Primary

   Secondary

   Higher

5. **Recent occupation**: ..................................................................................(complete it in your own words; if you are on disability pension or retirement pension, fill in: Disability Pension, Retirement Pension; if you are still partially active in your profession despite your disability pension/retirement pension, provide both pieces of information)
6. **Place of residence** (do not provide your address!) - fill in only the name of your town/city or village: ..............................................................................
7. **Marital status**: married

   single (choose from the following):

   Divorced

   Widowed

   Unmarried

8. **Number of people living with you in the household** (house, apartment), do not include yourself!:..............................................................
9. **Most recent weight** (kg):.......................................
10. **Latest height** (cm):....................................
11. **Do you have a certified (or any) degree of disability**:

    **YES**

    **NO**

12. **Do you use medical supplies <u>on a long-term basis</u>?**

    **YES***

**NO**

***Select from the following if you answered YES (*<u>multiple choice possible):</u>***

Wheelchair
Walker
Crutches
Walking stick
Artificial limb
Limb orthosis
Orthopedic or other special footwear
Spinal corset
Glucose monitoring system (<u>do not mark if</u> you use a glucose meter!)
Insulin pump
Pull-up pants
Stoma kit
Hearing aid
Catheters
Truss

**14. Do you have diabetes** (mark YES only if you have already been diagnosed with this disease)**?**

***YES**

**NO**

*****If you know the result of your last (max 6 months ago) **HbA1c** test, please provide it: **.........**

**15. Do you suffer from any of the following conditions** *(<u>you can mark more than one</u>)* **:**

I have had a heart attack

I have cardiac ischemia but have not had a heart attack

Atrial fibrillation

Heart rhythm disorders other than atrial fibrillation

I have suffered a stroke (colloquially, brain hemorrhage)

Hypertension

Varicose veins

Pulmonary embolism or venous thrombosis requiring long-term blood thinning treatment

Pulmonary or bronchial disease requiring long-term treatment

Disease of the gastrointestinal tract (stomach, intestines, liver, pancreas) requiring long-term treatment (such as reflux, ulcers, inflammation, diarrhea, constipation, insufficiency/failure, cirrhosis, steatosis)

Diseases of the kidneys, prostate gland, urinary bladder

Diseases of the musculoskeletal system (bones, muscles, joints) such as inflammation, degeneration, etc.

Cancer under treatment (any location)

Female reproductive system diseases (such as prolapse of the reproductive organs)

Skin diseases (such as psoriasis)

Anemia (erythrocytopenia)

Thyroid disease

Mental disorder (such as depression, neurosis, schizophrenia) - only if confirmed by a physician

Other disease, indicate which:.....................................................................................

16. **Are you taking medications on a long-term basis due to the diseases marked above**

(*do not mark if they are not prescribed*)

YES

NO

YES, BUT ONLY FOR SOME OF THE ABOVE DISEASES

**<u>Factors that limit regular physical activity</u>**

*Please circle the answer of your choice.*

1. **How would you rate your level of daily physical activity** (*<u>only one choice possible)</u>*
   A. Low
   B. Sufficient
   C. High

2. **Would you like to be more physically active** (*<u>more, more often) (only one choice possible)</u>*
   A. NO
   <u>(Note - this answer refers only to your desire, not lack of opportunity!)</u>

   B. YES*
   (Note - if you want to but cannot for some reason be more active, select YES)

   **If you selected NO in this question, leave the rest of the form without filling out!**

3. **Indicate the reasons why** (in your opinion) **your physical activity <u>cannot</u> be increased** *(<u>multiple choice possible</u>*).

   A. I do not have time - because of my primary job (recent occupation)

   B. I do not have time - for a reason other than work (recent occupation): select the reason(s) from the following:

   - taking care of children

   - caring for a family member or loved one other than children

   - additional non-physical activities that you cannot or do not want to abandon (such as charitable activities in an institution or independent charitable activities; activities in religious organizations, such as church; reading books; playing an instrument; playing cards/chess or other; crocheting/ knitting; painting or other activities that occupy your time but do not require physical exertion)

   C. I feel tired of the responsibilities I have to do on a daily basis (primary job, secondary duties) even though I would find time for physical activity.

   D. I have time but have no idea what I could do.

E. I do not have the conditions to undertake physical activity (small apartment, lack of centers like gym, lack of urban greenery such as a park).

F. *I have diseases that limit my physical activity and:

- my physician has prohibited me from undertaking physical exertion

- I think that exertion will cause me harm (this is my opinion)

G. I do not like physical activity but would like to do it for my health.

H. I can't find the motivation to do physical activity ("I do not want to") but I would like to do it for my health.

I. I would like to be physically active but I do not like (or I am afraid of) exercising alone, and I do not have anyone with whom to undertake such activity.

J. I do not have money and I think that undertaking additional physical activity requires an expense on my part.

*4.* *If you marked YES in item 2 - which means you would like to be more physically active - select from the following where you would prefer to undertake such activity *(you can mark both)*

- at home

- outside the home (park, gym, etc.)

5. *If you indicated that the diseases can limit physical activity (answer F in item 3), select them from the list below (*multiple choice possible*).
**Remember**- mark only those diseases or symptoms because of which **you think people cannot undertake** physical activity (do not mark a disease just because you have been diagnosed with it if it is not an obstacle to physical activity):

- heart disease

- respiratory (pulmonary, bronchial) disease

- shortness of breath/dyspnea (suffocation)

- edemas

- obesity/overweight

- joint disease (spine, hips, knees, feet)

- dizziness

- leg pain when walking (not sure why)
- diseases of the nervous system (person after stroke/brain hemorrhage, nerve palsy, other diseases of the nervous system such as multiple sclerosis)

- mental disorders (depression, schizophrenia or others treated by a psychiatrist)

- cancer (regardless of the location and the system it occupies)

- urinary incontinence

- *diabetes

- varicose veins

- leg/foot wound

- other disease than the above (also mark if you do not know if your disease belongs to one of the above groups). Please provide the medical name of this disease or write it in your own words:
…………………………………………………………………………………………………………………………………….

6. *If you marked diabetes as a disease because of which you think you cannot be more physically active answer the additional question:

   - I am treated with insulin

   - I am not taking insulin
