# Supplementary material for: The Level and Limitations of Physical Activity in Elderly Patients with Diabetes
Source: J Clin Med. 2024 Oct 23;13(21):6329. doi: 10.3390/jcm13216329 (PMC11546819; doi:10.3390/jcm13216329)
Supplement: Supplementary file 1 [file jcm-13-06329-s001.zip › Supplementary File S2.pdf]

Table S1. Results of the Accompanying Survey (AS).

Variable	Class	N	∑N	%	∑%
**Part A: demographic and epidemiological data**					
gender	men	78	78	37,68	37,7
	women	129	**207**	62,32	100
education	primary	26	26	12,56	12,56
	secondary	119	145	57,49	70,05
	higher	55	**200**	26,57	96,62
place of residence	city	180	9	0,48	4,35
	village	8	8	3,86	3,86
	small city	1	**189**	86,96	91,30
marital status	single	78	78	37,68	37,68
	in a relationship	123	**201**	59,42	97,10
number of people living in the household	0	8	8	3,86	3,86
	1	73	81	25,27	39,13
	2	83	164	40,10	79,23
	≥3	29	**193**	14,01	93,24
a certificate of disability	No	169	169	81,64	81,64
	Yes	35	**204**	16,91	98,55
use of medical supplies	No	155	155	74,88	74,88
	Yes	44	**199**	21,26	96,14
Do you chronically suffer from the following health problems? Q1_0	No	15	15	7,25	7,25
	Yes	192	**207**	92,75	100
IHD and MI Q1_1	No	186	186	89,86	89,86
	Yes	21	**207**	10,14	100
IHD no MI Q1_2	No	191	191	92,27	92,27
	Yes	16	**207**	7,73	100
AF Q1_3	No	174	174	84,06	84,06
	Yes	33	**207**	15,94	100
arrhythmias other than AF Q1_4	No	194	194	93,72	93,72
	Yes	13	**207**	6,28	100
stroke in the past Q1_5	No	192	192	92,75	92,75
	Yes	15	**207**	7,25	100
Hypertension Q1_6	No	51	51	24,64	24,64
	Yes	156	**207**	75,36	100
chronic venous disease (varices) Q1_7	No	161	161	77,78	77,87
	Yes	46	**207**	22,22	100
embolism or thrombosis requiring long-term treatment	No	202	202	97,58	97,58
	Yes	5	**207**	2,42	100

Q1_8					
lung or bronchial disease Q1_9	No	193	193	93,24	93,24
	Yes	14	**207**	6,76	100
gastrointestinal disease Q1_10	No	148	148	71,50	71,50
	Yes	59	**207**	28,50	100
diseases of the kidneys, prostate gland, urinary bladder Q1_11	No	160	160	77,29	77,29
	Yes	47	**207**	22,71	100
diseases of the musculoskeletal system Q1_12	No	127	127	61,35	61,35
	Yes	80	**207**	38,65	100
cancer under treatment Q1_13	No	200	200	96,62	96,62
	Yes	7	**207**	3,38	100
female reproductive system diseases Q1_14	No	125	125	96,90	96,9
	Yes	4	**129**	3,10	100
skin diseases Q1_15	No	194	194	93,72	93,72
	Yes	13	**207**	6,28	100
thyroid gland diseases Q1_16	No	165	165	79,71	79,71
	Yes	42	**207**	20,29	100
mental disorders Q1_17	No	200	200	96,62	96,62
	Yes	7	**207**	3,38	100
other chronic diseases Q1_18	No	189	189	91,30	91,30
	Yes	18	**207**	8,70	100
**Part B: self-assessment of physical activity level and preference of where to undertake it**					
How would you rate your level of physical activity?	Insufficient	84	84	43,98	43,98
	Sufficient	87	171	45,55	89,53
	High	5	**176**	2,62	92,15
Do you want to be more physically active?	No	63	63	32,98	32,98
	Yes	104	**167**	54,45	87,43
Where would you like to undertake additional physical activity?	at home	26	80	12,56	38,65
	outside the home	54	54	25,09	26,09
	place does not matter	12	**92**	5,80	44,44
**Part C: obstacles to undertaking physical activity and opinions on the possibility of undertaking it in chronic disease**					
I don't have time - because of my profession (work) Q2_1	No	95	95	95,96	95,96
	Yes	4	**99**	4,04	100
	No	72	72	72,73	72,73

I don't have time - for other reason than work Q2_2	Yes	27	**99**	27,27	100
I feel tired by the duties I have to do every day, even though I have time Q2_3[C]	No	77	77	77,78	77,78
	Yes	22	**99**	22,22	100
I have time but have no idea what I could do Q2_3[D]	No	77	77	77,78	77,78
	Yes	22	**99**	22,22	100
I don't have the conditions to undertake physical activity Q2_4	No	95	95	95,96	95,96
	Yes	4	**99**	4,04	100
I have diseases that limit my physical activity Q2_5	No	46	46	46,46	46,46
	Yes	53	**99**	53,54	100
I don't like physical activity, but I would like to undertake it Q2_6	No	91	91	91,92	91,92
	Yes	8	**99**	8,08	100
I can't find the motivation to do physical activity (I don't want to), but I would like to do it for my health Q2_7	No	70	70	70,71	70,71
	Yes	29	**99**	29,29	100
I would like to be physically active, but I don't like to exercise alone (and I don't have anyone to exercise with), or I am afraid to exercise alone Q2_8	No	68	68	68,69	68,69
	Yes	31	**99**	31,31	100
I don't have money to undertake physical activity, and in my opinion, it requires expenses Q2_9	No	97	97	97,98	97,98
	Yes	2	**99**	2,02	100
Do you think that the following disease (or symptoms) may be an obstacle to physical activity? Q3_0	No	93	93	44,93	44,93
	Yes	114	**207**	55,07	100
heart disease Q3_1	No	185	185	89,37	89,37
	Yes	22	**207**	10,63	100
respiratory disease Q3_2	No	196	196	94,69	94,69
	Yes	11	**207**	5,31	100
Oedema Q3_3	No	191	191	92,27	92,27
	Yes	16	**207**	7,33	100

obesity, overweight Q3_4	No	182	182	87,92	87,92
	Yes	25	**207**	12,08	100
joint disease Q3_5	No	137	137	66,18	66,18
	Yes	70	**207**	33,82	100
Dizziness Q3_6	No	178	178	85,99	85,99
	Yes	29	**207**	14,01	100
leg pain Q3_7	No	168	168	81,16	81,16
	Yes	39	**207**	18,84	100
nervous system diseases Q3_8	No	204	204	98,56	98,56
	Yes	3	**207**	1,45	100
mental disorders Q3_9	No	205	205	99,03	99,03
	Yes	2	**207**	0,97	100
urinary incontinence Q3_10	No	181	181	87,44	87,44
	Yes	26	**207**	12,56	100
Diabetes Q3_11	No	139	139	67,15	67,15
	Yes	68	**207**	32,85	100
varicose veins Q3_12	No	193	193	93,24	93,24
	Yes	14	**207**	6,76	100
leg wounds Q3_13	No	206	**206**	99,52	99,52
	Yes	1	**207**	0,48	100
other diseases Q13_14	No	200	200	96,62	96,62
	Yes	7	**207**	3,38	100
Dyspnoea Q13_15	No	184	184	88,89	88,89
	Yes	23	**207**	11,11	100
cancer disease Q13_16	No	204	204	98,55	98,55
	Yes	3	**207**	1,45	100

N- number of respondents; ∑N- cumulative N; ∑%- cumulative percentage; DM-diabetes mellitus, IHD- ischemic heart disease; MI- Myocardial infarction; AF- atrial fibrillation, Q-question (helpful in relation to Figure no. 4)
